# Poly(ionic liquid) Based Composite Electrolytes for Lithium Ion Batteries

**DOI:** 10.3390/polym13244469

**Published:** 2021-12-20

**Authors:** Robert Löwe, Thomas Hanemann, Tatiana Zinkevich, Andreas Hofmann

**Affiliations:** 1 Institute for Applied Materials, Karlsruhe Institute of Technology, Hermann-von-Helmholtz-Platz 1, D-76344 Eggenstein-Leopoldshafen, Germany; robert.loewe@kit.edu (R.L.); thomas.hanemann@kit.edu (T.H.); tazink1984@gmail.com (T.Z.); 2 Department of Microsystems Engineering, University of Freiburg, Georges-Köhler-Allee 102, D-79110 Freiburg, Germany

**Keywords:** lithium ion batteries, polymerizable ionic liquids, poly(ionic liquid)s, composite polymer electrolytes, ionogel electrolyte, lithium diffusion, PFG-NMR, MPPyrr-TFSI, EC

## Abstract

Polymerized ionic liquids (PIL) are an interesting substance class, which is discussed to transfer the outstanding properties and tunability of ionic liquids into a solid material. In this study we extend our previous research on ammonium based PIL and discuss the influence of additives and their usability as polymer electrolyte membranes for lithium ion batteries. The polymer electrolyte is thereby used as replacement for the commercially widespread system of a separator that is soaked with liquid electrolyte. The influence of the material composition on the ionic conductivity (via electrochemical impedance spectroscopy) and the diffusion coefficients (via pulsed-field-gradient nuclear magnetic resonance spectroscopy) were studied and cell tests with adapted membrane materials were performed. High amounts of the additional ionic liquid (IL) MPPyrr-TFSI (1-methyl-1-propylpyrrolidinium bis(trifluoromethylsulfonyl)imide) increased the ionic conductivity of the materials up to 1.3·10^−4^ S·cm^−1^ but made the usage of a cross-linker necessary to obtain mechanically stable membranes. The application of liquid electrolyte mixtures with ethylene carbonate (EC) and MPPyrr-TFSI decreased ionic conductivity values down to the 10^−9^ S·cm^−1^ range, but increased ^7^Li diffusion coefficients with increasing amounts of EC up to 1.7·10^−10^ m^2^·s^−1^. Cell tests with two membrane mixtures proofed that it is possible to build electrolyte membranes on basis of the polymerized ionic liquids, but also showed that further research is necessary to ensure stable and efficient cell cycling.

## 1. Introduction

**Figure 1:**
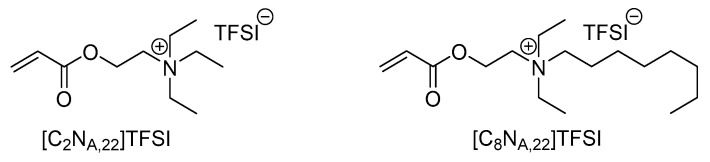
Structures of the monomeric ionic liquids, which were selected to build up a polymer electrolyte membrane for lithium ion batteries.

Increasing safety of lithium ion battery cells is a major concern in the field of battery research [1,2,3,4]. Ionic liquids and farther polymerized ionic liquids may be a saver alternative to conventional separator–electrolyte systems due to their outstanding properties like high thermal and electrochemical stability as well as low flammability and extremely high tuneability by varying the cationic or anionic structure [5,6,7,8]. Previously, we presented the synthesis and structure-property relationships of eight new, polymerizable ionic liquids (IL) and their application in polymer electrolyte membranes with lithium bis(trifluoromethanesulfonyl)imide (LiTFSI) as conducting salt for lithium ion batteries [9,10]. It was found that the ionic conductivity values of the membranes based on polymerized ionic liquids (PIL) are not sufficient for the usage in battery cells. Concerning Long et al. an ionic conductivity value of more than 10^−4^ S·cm^−1^ at room temperature is a basic requirement for polymer electrolytes, whereas the studied polymer mixtures featured ionic conductivity values in the magnitude of 10^−9^ S·cm^−1^ to 10^−8^ S·cm^−1^ at 25 °C [10,11]. Anyway, it turned out that the polymerizable ionic liquids shown in Figure 1 have the highest potentials for further optimization by tuning the membranes properties with additives.

On one hand, the longer alkyl chain in the octyl derivate *N*-[2-(acryloyloxy)ethyl]-*N,N-*diethyl-*N*-octylammonium bis(trifluoromethanesulfonyl)imide ([C_8_N_A,22_]TFSI) leads to higher ionic conductivity values of the resulting polymer membranes, compared to the ethyl derivate [C_2_N_A,22_]TFSI [10]. On the other hand, the longer alkyl chain is accompanied with the reduction of mechanical stability of the membranes. Therefore, the ethyl derivate *N*-[2-(acryloyloxy)ethyl]-*N,N,N*-triethylammonium bis(trifluoromethanesulfonyl)imide ([C_2_N_A,22_]TFSI) may be an interesting candidate with beneficial mechanical properties [10].

To improve the polymer electrolytes properties in context of a battery application, the introduction of additives is often mentioned. As the literature shows, the addition of a non-polymerizable ionic liquid to polymer electrolyte systems can improve ionic conductivity values up to the magnitude of 10^−3^ S·cm^−1^ at room temperature [12]. Moreover, Tian et al. describe the appearance of self-healing properties when high amounts of up to 45 wt% of *N*-methyl-*N*-propylpyrrolidinium-TFSI (MPPyrr-TFSI) were added to a cationic TFSI-PIL [13]. Besides the addition of pure ionic liquids as liquid electrolyte content, organic solvents can be added. Mixtures of IL with organic carbonates have a lower risk of fire than the pure organic solvents, as shown by Lombardo et al. [14]. The IL MPPyrr-TFSI was discussed to feature advantageous electrochemical properties like a wider electrochemical windows over similar ionic liquids and can also be used in combination with ethylene carbonate while keeping high electrochemical stability and ionic conductivity values [15].

The addition of inorganic particles to the polymer matrix is a well-known method in the field of polyethylene oxide (PEO) electrolytes in order to have a beneficial effect on the material properties [16,17]. The resulting composite polymer electrolytes provide higher performance in terms of mechanical stability, ionic conductivity, capability of electrolyte absorption and stability at the interface between polymer and metallic lithium [16,18,19,20,21]. The higher ionic conductivity values result primarily from the reduction of crystallinity or the formation of amorphous centers in the PEO polymer, which also results in a better conduction of the lithium ions [16,22,23]. In a review article Meng et al. summarize that the particle size of inactive, ceramic particles has an influence on the ionic conductivity and lithium migration through a composite polymer electrolyte [24]. It is described that the lithium transport takes place via the segment mobility of the polymer on the one hand and along the interface between the particles and the polymer on the other hand. A larger particle surface should therefore lead to a larger interface of the particles with the polymer matrix and result in a higher contribution to the lithium transport by the latter mechanism. Accordingly, it can be assumed that smaller ceramic particles in the polymer matrix should lead to a better lithium ion mobility. This increase in ion conductivity by adding smaller particles was also found in amorphous PEO phases, which indicates that this mechanism is not directly or exclusively dependent on the structural change in the polymer or the reduction of crystallinity caused by the added particles [24]. Further, the addition of nanoparticles influences the ionic conductivity in amorphous polyester- and polycarbonate-based polymer electrolytes [25]. Correspondingly, this effect could also play a role in amorphous, polymerized ionic liquid systems. Preliminary studies revealed that sedimentation of the particle agglomerates is a serious issue [26]. Therefore, the usage of surface modified particles, which feature a polymerizable function, is focused.

Furthermore, the membrane properties can be adjusted by adding cross-linking agents before the polymerization. The addition of small amounts of cross-linkers to PIL membranes can lead to a significant increase in the mechanical and thermal stability without negatively affecting the ionic conductivity [27,28]. Washiro et al. describe that the addition of cross-linkers to poly-ionic liquids leads to flexible, free-standing membranes instead of sticky, rubber-like materials without this additive [29].

In this study the incorporation of additives from different classes (inorganic filler, cross-linker, ionic liquid, carbonate electrolyte) into the polymerized ionic liquid network was investigated. The property adjustment was carried out in a systematical manner with the objective to develop an electrolyte membrane with reasonable characteristics for lithium ion batteries. Since the polymeric membrane replaces the typically used separator—liquid electrolyte system, it must be mechanically stable as well as sufficiently capable of transferring the lithium ions through the membrane. Primarily used analytical methods were electrochemical impedance spectroscopy (EIS), pulsed-field-gradient nuclear magnetic resonance spectroscopy (PFG-NMR) and cell tests in coin cells with the finally chosen polymer electrolyte membranes.

## 2. Materials and Methods

### 2.1. Materials

Monomers of the polymerizable ionic liquids *N*-[2-(Acryloyloxy)ethyl]-*N,N,N*-triethylammonium bis(trifluoromethanesulfonyl)imide ([C_2_N_A,22_]TFSI) and *N*-[2-(Acryloyloxy)ethyl]-*N,N-*diethyl-*N*-octylammonium bis(trifluoromethanesulfonyl)imide ([C_8_N_A,22_]TFSI) were synthesized as described in reference [9]. LiTFSI as conducting salt (99.95% on metal basis) and the initiator Irgacure651 were purchased from Sigma-Aldrich (Munich, Germany) and Ciba Chemicals (Basel, Switzerland) and used as received. Acetone (99.9%; anhydrous over molecular sieve 3 Å) was purchased from Acros Organics (Geel, Belgium). As nanoparticle fillers Aerosil R7200 with primary particle sizes of approximately 20 nm from Evonik (Essen, Germany) was chosen. The filler consists of fumed silica particles, which are surface modified with a methacryl silane and therefore feature a polymerizable methacrylic function. Di(trimethylolpropane) tetraacrylate as a cross-linker was purchased from Sigma-Aldrich (Munich, Germany). Ethylene carbonat (EC; ≥99.8%; recrystallized over molecular sieve 3 Å) and *N*-methyl-*N*-propylpyrrolidinium-TFSI (MPPyrr-TFSI; 99%) were purchased from Huntsman (The Woodlands, TX, USA) and IoLiTec (Heilbronn, Germany), respectively. Electrode foils with the active materials lithium nickel manganese cobalt oxide (NMC-111, 2 mAh·cm^−2^), graphite (2 mAh·cm^−2^), lithium iron phosphate (LFP, 1 mAh·cm^−2^) and lithium titanate (LTO, 1 mAh·cm^−2^) were purchased from Custom Cells (Itzehoe, Germany). Metallic lithium (99.9%) as electrode material was purchased as foil from Alfa Aesar (Kandel, Germany).

### 2.2. Polymerisation Equipment

Polymerization of the monomer mixtures was carried out in an *EC-500* UV chamber (Electro-lite, Bethel (CT), USA), equipped with four 9 W UV lamps peaking at 365 nm (P/N 82469; Electro-lite, Bethel, AK, USA). The UV-chamber was located inside an argon-filled glovebox (H_2_O, O_2_ < 0.5 ppm) to prevent water contamination.

### 2.3. Preparation of the PIL-Based Electrolyte Membranes

The preparation of polymer electrolyte membrane layers follows a routine that can be divided into three working steps:

- Preparation of a mixture with the IL-monomers and all additives
- Application of the monomer slurry onto an electrode foil for cell tests or an aluminum foil for electrochemical impedance spectroscopy (EIS)
- Polymerization of the monomers under UV radiation

All steps were carried out under inert conditions in an argon-filled glovebox (H_2_O, O_2_ < 0.5 ppm). The mixtures were freshly prepared before polymerization. The conductive salt content was set to 20 mol% and the initiator content to 1 mol%, which means 0.2 mol LiTFSI and 0.01 mol Irgacure651 per 1 mol of the IL-monomer, respectively. The particle content was 5 wt% of Aerosil R7200 measured against the total polymer electrolyte mass. The amount of liquid electrolyte additives, which means MPPyrr-TFSI, EC and their mixtures was set to 45 wt % of the total polymer electrolyte mass.

### 2.4. Preparation of the Monomer Mixture

First, the conductive salt LiTFSI (20 mol%), Aerosil R7200 (5 wt %) and MPPyrr-TFSI were weighed together into an amber glass bottle. The IL-monomers, which were stored in acetone (50 wt % IL content), were added using a plunger lift pipette. The mixture was dispersed for 30 s with an ULTRA-TURRAX T8 dispersing rod (IKA, Staufen im Breisgau, Germany) and then stirred under vacuum for 45 min at 35 °C to remove the acetone. Then, the initiator Irgacure651, the ethylene carbonate (EC) and the cross-linking agent bis(trimethylol)propane tetraacrylate (TMPTA) were added. The mixture was stirred again under vacuum for 15 min.

### 2.5. Processing of the Monomer Mixtures to Electrolyte Membranes

**Figure 2:**
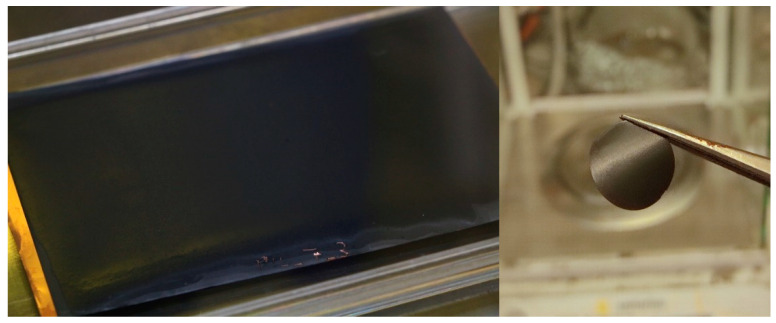
Polymer electrolyte layer on graphite electrode directly after polymerization (**left**) as well as a punched-out membrane on a LiNi_1/3_Mn_1/3_Co_1/3_O_2_ electrode for tests in a coin cell (**right**).

The monomer mixtures were polymerized directly onto the surface of the electrode that was used. Therefore, the electrode or aluminum foil were fixed on a plain glass plate. A thin layer of the monomer mixture was applied using a doctor blade with a fixed gap of 200 μm. The doctor blade was filled with the monomer mixture and drawn slowly (approximately 15 cm·min^−1^) over the electrode sheet. Due to the relatively low viscosity of the monomer slurries, the suspensions did spread out on the electrode surfaces. Therefore, the final monomer layer thickness is well below 200 µm. The glass plate with the coated electrode sheet was immediately placed within the UV-chamber and exposed for 40 min. Figure 2 shows an example of a graphite sheet coated with composite polymer electrolyte in the format of approximately 8 × 12 cm and a coated, punched-out NMC electrode with a diameter of 16 mm.

### 2.6. Measurements

Electrochemical impedance spectroscopy (EIS) of the polymer electrolyte layers on aluminum foil, dynamic scanning calorimetry (DSC) and PFG-NMR measurements were executed as described in our previous publication and are specified in the supplementary information (SI-1), as well [10]. Exemplary data plots for DSC, EIS and PFG-NMR measurements are given in the Supporting Materials (SI-2, Figures S1–S4). Cyclic voltammetry was carried out on a *Zennium* potentiostat (ZAHNER-elektrik, Kronach, Germany). For this purpose, the electrolyte layers polymerized on aluminum foil were installed in Swagelok cells against metallic lithium electrodes and analyzed at 25 °C between 2 and 6 V with a rate of 1 m·Vs^−1^.

Cross-sections through material layers for the purpose of scanning electron microscopy (SEM) were created by the ion beam etching system *EM TIC 3X* (Leica Microsystems, Mannheim, Germany) using argon ions. The SEM images were recorded on a *Zeiss Supra 55 VP* microscope (Carl Zeiss, Oberkochen, Germany).

For cell tests, electrodes with a diameter of 16 mm were used, with the exception of metallic lithium, which was used with a diameter of 12 mm. Since the metallic lithium was not coated with the polymer electrolyte which also works as the separator, the lithium anode necessarily needs to be of a smaller diameter than the polymer-coated cathodes to prevent a short circuit. Further, within the coin cell preparation process, the stack is pressed and the lithium flattened. The coated anode and cathode materials (with exception of lithium metal, which was not coated) were placed directly one on top of the other in the coin cells. Stainless steel plates were used as a spacer and a small spring was used to ensure contact with the cell housing. The cells were closed with a *CR100* coin cell crimper (UNB, Fredericton, NB, Canada) at 1000 psi (approx. 69 bar). The cells were cycled in various test programs with different charging and discharging speeds at 25 and 70 °C, respectively. The cycling was carried out on *Liccy* cell cyclers (self-built by the Karlsruhe Institute of Technology, Karlsruhe, Germany) within an *IPP30plus* climatic chamber (memmert, Schwabach, Germany) for each temperature.

## 3. Results and Discussion

Within this section, the IL monomers are labeled in accordance to Figure 1. Materials with the polymerized ionic liquids are labeled with the prefix P, like P[C_2_N_A,22_]TFSI for the polymerized form of the [C_2_N_A,22_]TFSI monomer. The prefix P- is also used to describe that an electrode is coated with a PIL composite membrane, like P-NMC for a coated NMC electrode.

### 3.1. Influence of Additional Ionic Liquid and Cross-Linker on the Membrane Properties

Achieving reasonable ionic conductivity values of the polymer electrolyte membranes is seen as the first step for the optimization towards a usability within battery cells. As literature implies high concentrations of MPPyrr-TFSI (3.1·10^−3^ S·cm^−1^ at 25 °C [30]) as a reasonable additive this IL was used as a liquid electrolyte content within the polymer matrix [13]. It was found that membranes without an additional organic cross-linker was not able to fulfil the requirements regarding stability and mechanical resilience (see Supporting Materials, SI-3).

In order to solve this issue different concentrations of cross-linkers were investigated as an additive. The cross-linker is supposed to covalently connect the polymer chains during the polymerization to increase the mechanical stability of the polymer network [31]. To increase the mechanical strength while not significantly change the composition of the system, the tetraacrylate linker TMPTA was selected. Three cross-linker concentrations of 10, 5 and 2.5 mol% in respect to the monomer and both PIL monomers [C_2_N_A,22_]TFSI and [C_8_N_A,22_]TFSI were used for this study. Mixtures without the tetraacrylate linker were produced as a reference. Unfortunately, it was not possible to produce EIS spectra of the reference layer of P[C_8_N_A,22_]TFSI since the polymerized mixture behaved less than a polymer membrane and more than a highly viscous fluid. The results of the EIS and DSC measurements are shown in Table 1. The compositions of the PIL membranes are given in the Supporting Materials (SI-4, Table S1).

The measurements show that a higher amount of cross-linker leads to lower ionic conductivity values. Nevertheless, the values are still within the same order of magnitude as for the membranes without cross-linker and significantly higher than without liquid electrolyte content (10^−9^ S·cm^−1^ to 10^−8^ S·cm^−1^ at 25 °C [10]). This decrease in ionic conductivity is expected because the cross-linker makes the polymer matrix more rigid and less flexible, which slows down the ion transport within the matrix. For the membranes with P[C_2_N_A,22_]TFSI as well as P[C_8_N_A,22_]TFSI, it was found that a cross-linker content of 2.5 mol% is sufficient to produce mechanically sufficient stable layers. A higher content of the cross-linker only leads to lower ionic conductivity values without an additional benefit. The determined glass transition temperatures of all prepared materials show no significant trend and should not be over-interpreted. Due to the high amount of non-polymerized additional components, the glass transition appears to occur in a very wide temperature window. Therefore, the given values are more kind of an approximate value than a sharp glass transition temperature.

**Figure 3:**
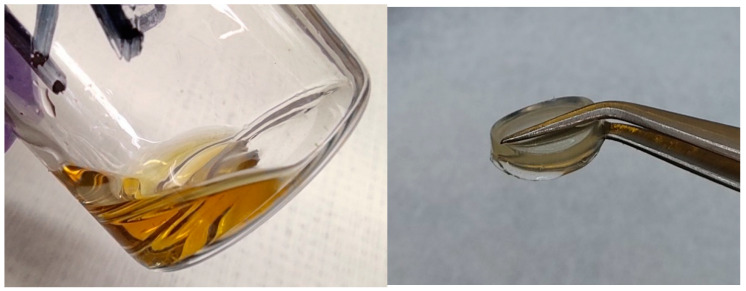
P[C_8_N_A,22_]TFSI polymer mixture with additional 45 wt % MPPyrr-TFSI and without cross-linker (**left**) same P[C_8_N_A,22_]TFSI polymer mixture (PIL_6_7) with additional 2.5 wt % of cross-linker (**right**).

In accordance with the previous investigations of the pure PIL-LiTFSI mixtures, the membranes with P[C_8_N_A,22_]TFSI feature higher ionic conductivity values than the ethyl derivatives [10]. It appears, that the ionic conductivity value of the P[C_8_N_A,22_]TFSI sample with 10 mol% cross-linker content is higher than of the P[C_2_N_A,22_]TFSI layer with 2.5 mol% cross-linker content at room temperature. As the fraction of cross-linker decreases, the ionic conductivity values increase, so that the mechanically stable P[C_8_N_A,22_]TFSI membrane with 2.5 mol% tetraacrylate content has a higher ionic conductivity than the mechanically unstable ethyl equivalent without cross-linker. Therefore, in terms of the optimization of the ionic conductivity, P[C_8_N_A,22_]TFSI seems to be the most suitable PIL basis. The influence of the cross-linker on the mechanical appearance is shown in Figure 3.

On the one hand, the photographic images show a P[C_8_N_A,22_]TFSI polymer mixture with 45 wt % of additional IL (MPPyrr-TFSI) without the cross-linker being added. The material appears as a high viscous liquid which does not feature sufficient mechanical strength to work as an ion conducting separator. On the other hand, the same polymerized mixture, but with additional 2.5 wt % cross-linker content occurs as a solid polymer membrane. Unfortunately, we were only able to produce this type of free-standing membranes in very thick bulks (several millimeters), which are not useful in battery systems. Thin polymer layers were polymerized directly onto the electrodes or in case of samples for EIS on the aluminum surface.

**Figure 4:**
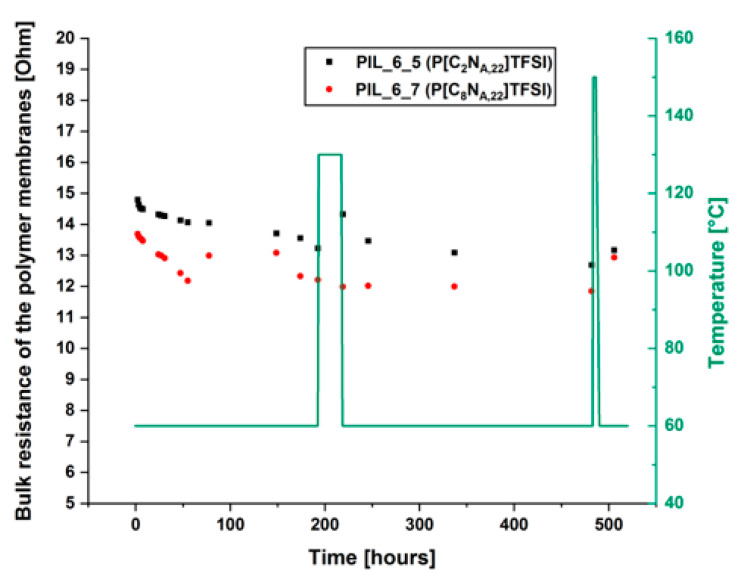
Change of the composite electrolyte membranes bulk resistance under thermal stress. Post-mortem membrane thicknesses of the samples are 99 μm for PIL_6_5 and 61 μm for PIL_6_7.

For use in a battery cell, it is essential to ensure that the electrolyte membrane is mechanical stable over time, otherwise a short-circuit could induce a thermal runaway. Therefore, a test procedure that investigates the mechanical behavior of the membranes indirectly by using EIS was introduced. The aim of this investigation was, to find out whether the membrane remains mechanically stable over a long period of time in a realistic battery scenario. For this purpose, samples of the P[C_2_N_A,22_]TFSI and P[C_8_N_A,22_]TFSI membranes with 2.5 mol% cross-linking agent were built into Swagelok cells and hold at 60 °C in a climatic chamber for 21 days. Within this period, the cells were heated up to 130 °C and kept at this temperature for 24 h. This temperature was chosen because commercial polyethylene (PE) membranes melt at 130 °C [32]. Furthermore, the cells were kept at 150 °C for 3 h. Higher temperatures could not be applied to prevent the polypropylene parts of the Swagelok cells from melting. The electrochemical impedance spectra of the membranes were recorded in regular intervals at 60 °C and the bulk resistances of the polymer layers were determined. Figure 4 shows the bulk resistance and applied temperature program over the time.

Overall, the membranes were exposed to high thermal stress during the test, which should not occur under normal battery operation. The data show that the measured bulk resistances decrease slightly over time. The decrease in resistances is mainly assumed to be the result of two factors: (i) the decrease in the layer thickness due to the polymer membranes being pushed out of the gap between the electrodes (ii) the diffusion of atmospheric water into the Swagelok cell during the time at which the cell is held at 60 °C. It was proven by Karl-Fischer titration that the membranes are getting contaminated with water when the Swagelok cells are stored at atmospheric conditions for several days (further information available in the Supporting material section). Even after the treatments at very high temperatures, there is no significant change in the bulk resistance. This shows that the electrolyte membranes do not melt or soften in such a way that a short circuit occurs, even in extreme thermal stress situations. After heating the cells up to 150 °C, the resistances were increasing again. It can therefore be assumed that the decrease in resistance was at least partly observed due to water contaminations. During the heating, the water was displaced from the membranes again. Summarizing, the results show, that the layers are mechanically very stable, even under significantly increased temperatures and are beneficial over PE membranes within this context.

### 3.2. Investigation of EC/MPPyrr-TFSI Mixtures as Liquid Electrolyte

The membrane mixture of P[C_8_N_A,22_]TFSI with 20 mol% LiTFSI, 5 wt % Aerosil R7200 particles, 45 wt % MPPyrr-TFSI and 2.5 wt % tetraacrylate delivers sufficiently high ionic conductivity and thermal stability. Therefore, the mixture was used as polymer electrolyte in coin cells with NMC against graphite as well as NMC against metallic lithium. Thereby, it was found that the cells do cycle properly, but experience a kind of cell defect without breakdown of the voltage. Typically, slow C-rates are used for the formation of a stable SEI-layer within the first cycles [33,34] and should promote stable cycling even if the lithium ion transfer is relatively slow. However, even at an elevated temperature of 50 °C and slow charging rates of C/20, stable cycling was not possible.

Two effects were assumed to cause this behavior: (i) the suppression of a SEI formation and occurring side reactions which damage cell components; (ii) an insufficient transfer of lithium ions through the membrane and/or through the boundary interface of active materials and polymer electrolyte. To solve these issues, the MPPyrr-TFSI as liquid electrolyte was partly exchanged against different amounts of EC. During the first cycles EC is supposed to form a stable, almost ideal SEI layer [35,36,37]. Furthermore, the literature shows a high electrochemical stability and ionic conductivity for electrolyte mixtures of MPPyrr-TFSI and ethylene carbonate [15]. The influence of the ratio of MPPyrr-TFSI to ethylene carbonate on the mobility of lithium ions was studied by PFG-NMR spectroscopy, which delivers the diffusion coefficients of the ^7^Li ions. Table 2 shows the determined properties of P[C_8_N_A,22_]TFSI polymer electrolyte membranes with varying ratios of MPPyrr-TFSI to EC and a comparison to P[C_2_N_A,22_]TFSI for one sample. The total amount of additional liquid electrolyte, represented by EC and MPPyrr-TFSI, was held constant and adds up to 45 wt% of the overall material mass. Although the pure ethylene carbonate is solid at room temperature, it is termed as liquid electrolyte in our context since there are no signs of a crystallized component in the final products at room temperature. All polymer mixtures appear as colorless and transparent membranes at room temperature and the DSC measurements indicate that there are no crystallized phases above room temperature.

In terms of the glass transition temperature *T_g_*, no significant influence of the electrolyte composition was found up to a MPPyrr-TFSI: EC ratio of 50:50. At higher EC contents, melting points arise in the DSC data. At room temperature and above the appearance of crystallization phenomena may not influence the materials suitability for a battery usage since the melting point are below room temperature, whereas the one of neat EC is 36.2 °C [38]. Below the crystallization temperatures, the lithium ion transport could be strongly limit, since the liquid EC content as major transport phase is not assisting the ion transport and may even immobilize the conducting salt partly by complexation within the solidified phase.

**Figure 5:**
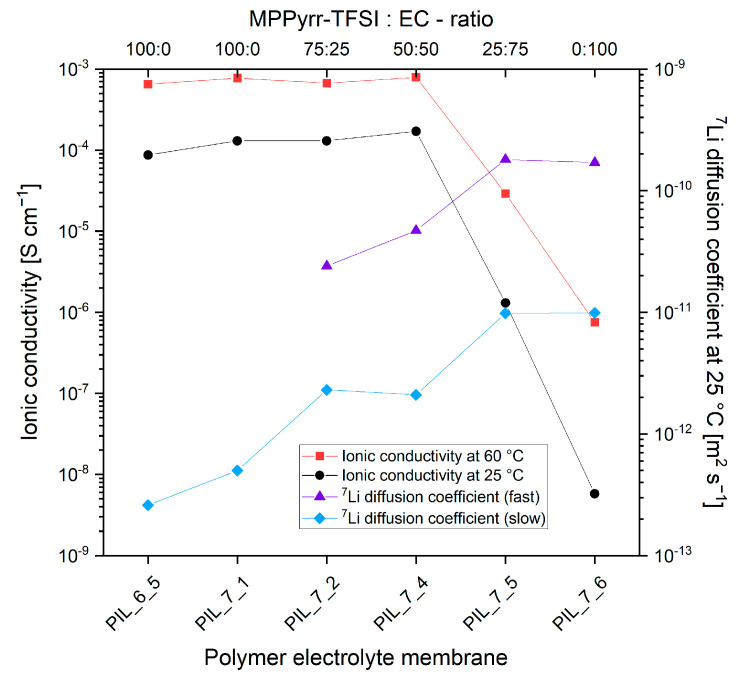
Dependence of the ionic conductivity values and ^7^Li diffusion coefficients at 25 °C on the monomer structure and the composition of the liquid electrolyte. The two appearing ^7^Li diffusion coefficients in case of ethylene carbonate containing materials are labeled as slow and fast. The points were connected for better visualization. Standard deviation of the ionic conductivity values are 17.2% and the systematic error is 2.7%. Error of the measurement is 5.6%.

The influence of the liquid electrolyte composition on the ionic conductivity values and ^7^Li diffusion coefficients are presented graphically in Figure 5.

First of all, it is seen that the P[C_8_N_A,22_]TFSI material outperforms the P[C_2_N_A,22_]TFSI membrane not only in terms of ionic conductivity but also in terms of a better lithium ion mobility as the PFG-NMR results reveal. Therefore, the use of the [C_8_N_A,22_]TFSI monomer is seen beneficial over derivates with shorter alkyl side chains. Further, the data clearly show that the lithium diffusion becomes faster with increasing amounts of ethylene carbonate. This trend continues even above an electrolyte mixture ratio of 1:1 when the ionic conductivity values are decreasing drastically and reach magnitudes of the solvent free polymer membranes. By replacing MPPyrr-TFSI with ethylene carbonate, the ion density in the material is significantly reduced. A reduction in ionic conductivity values can be expected. The decrease down to the range of 10^−9^ S·cm^−1^ at 25 °C indicates that the increase in ionic conductivity through the usage of MPPyrr-TFSI is mainly due to the dissociated ions of the IL and not due to an improved mobility of the conductive salt ions.

Another observation was the emergence of a second, faster lithium diffusion coefficient when ethylene carbonate was added. This phenomenon can have various causes. It is conceivable that the addition of EC promotes the formation of different lithium complexes, which have different transport mechanisms through the membrane with different mobility. However, this approach does not provide any information about why the diffusion constants increase successively as a function of the EC concentration. Changing complex compositions could possibly play a role. However, it is an insufficient explanation for the fact that exactly two diffusion coefficients occur with increasing values as the EC content increases. It is assumed as much more likely explanation, that the first complex environment of the lithium ions is not influential for the increase in lithium mobility. Rather, different spatial influences for the two differently fast ions are assumed. The slower ions may be trapped in the polymer structure, from which the low mobility arises. The faster ions may be located in a liquid phase, which is partially decoupled from the polymer matrix. A corresponding inhomogeneity is not to be expected from the addition of pure MPPyrr-TFSI due to the ionic interactions between polymer and IL ions, as it has been described in the literature [13]. With this assumption, the slower diffusion coefficient would correspond to the movement of the lithium ions within the actual polymer matrix. The newly added diffusion constant is based on the lithium ions which are dissolved in a liquid ethylene carbonate phase. The conclusion of this assumption is that a non-ionic liquid electrolyte component (in our case EC) is necessary to enable sufficient lithium mobility for the use of the materials in battery cells.

### 3.3. Polymerization on Electrode Foils

**Figure 6:**
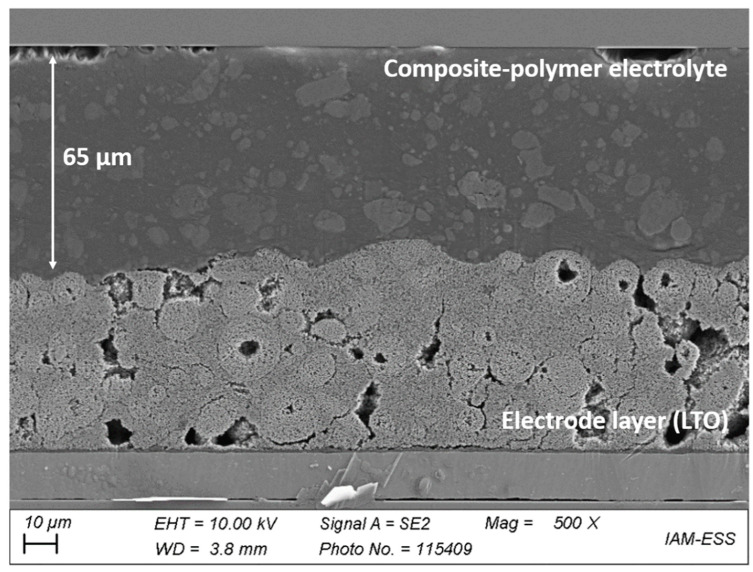
Scanning electron microscope image of the cross-section of a polymer electrolyte coated LTO electrode; 500× magnification.

The production of freestanding polymer membranes did not result in qualitatively satisfying layers. Therefore, the monomer mixtures were applied onto the electrode foils and polymerized directly on the active material surface. The electrode foils were bought from a commercial producer to exclude influences of a lab scale electrode preparation. To the best of the authors knowledge, the direct UV-polymerization onto an electrode surface was not described yet in literature, but may offer different advantages in production processes like the roll-to-roll process or in 3D printing. Figure 6 shows a cross-section of a LTO electrode foil on aluminum, which was coated with a PIL_7_6 monomer mixture and polymerized as described in Section 2. As the image shows, the resulting membrane features a plane surface and compensates unevenness of the active particle layer.

### 3.4. Cell Cycling

From the PFG-NMR results, it can be derived that a higher EC content could lead to better cell performance due to higher lithium ion mobility, even at lower ionic conductivity values. An ionic conductivity of at least 10^−4^ S·cm^−1^ at room temperature, as the literature claims, is therefore not a sufficient characteristic for the studied PIL materials to ensure sufficient lithium ion mobility. Moreover, it is necessary to ensure a sufficient mobility of the lithium ions and the compatibility of the polymer electrolyte materials with the electrodes. For this purpose the P[C_8_N_A,22_]TFSI membranes with 50% EC (PIL_7_4) and 100% EC (PIL_7_6) were polymerized directly on the active material foils (except for lithium metal) and cycled as full cells in a coin cell format. Two electrode combinations were used:

- NMC against metallic lithium; voltage range: 3.0–4.3 V
- LFP against LTO; voltage range: 1.0–2.5 V

Graphite was not used to avoid possible intercalation of TFSI ions in the electrode, which may damage the anode [15]. From a calculative point, metallic lithium is the most preferable anode material to achieve a maximized volumetric energy density [39]. Polymerization of the electrolyte material on lithium metal was tested successfully but turned out to be unpractical in further processing steps, like stamping out of the electrodes.

**Figure 7:**
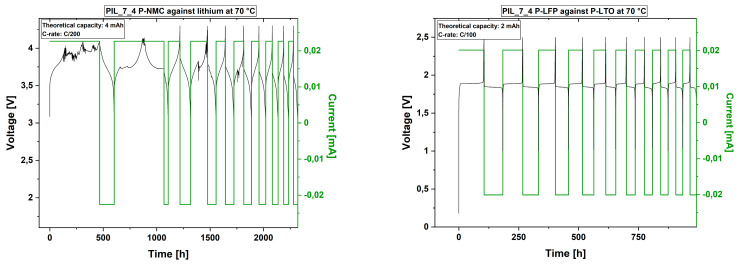
First 10 cycles of the cell test of the polymer electrolyte with MPPyrr-TFSI to EC ratio of 1:1 (PIL_7_4) at 70 °C with the electrode combinations NMC111 against lithium (**left**; C/200) and lithium iron phosphate against lithium titanate (**right**; C/100). Images including all executed cycles are given in the Supporting Material section (Figure S5).

Cell tests of both polymer electrolyte materials with NMC against lithium metal did not deliver stable cycles neither at 25 °C nor at 70 °C. Even with extremely slow C-rates of C/200, cell defects in terms of a total loss in capacity occurred after several cycles. Figure 7 shows exemplarily how the capacity decreases strongly within the first cycles. The same phenomenon was found for the usage of LFP against LTO as electrode materials.

Although the C-rate was faster with the LFP-LTO electrode combination, the cell stability appeared to be superior over NMC-lithium metal. Therefore, two main issues were suggested, (i) an insufficient lithium transference, which support (ii) unwanted side reactions due to the resulting electrode polarization. Due to the higher voltage within the NMC system, the side reactions may proceed faster and hinter the lithium ion transport or even consume lithium ions in an irreversible matter. Although, the data show a lack in performance, it is also seen, that the polymer membranes did not mechanically fail, since no short circuit occurred. After approximately 125 days, the cell cycling of the NMC-lithium cell was stopped without any unexpected potential drop. In terms of safety, the membranes therefore feature desirable properties.

**Figure 8:**
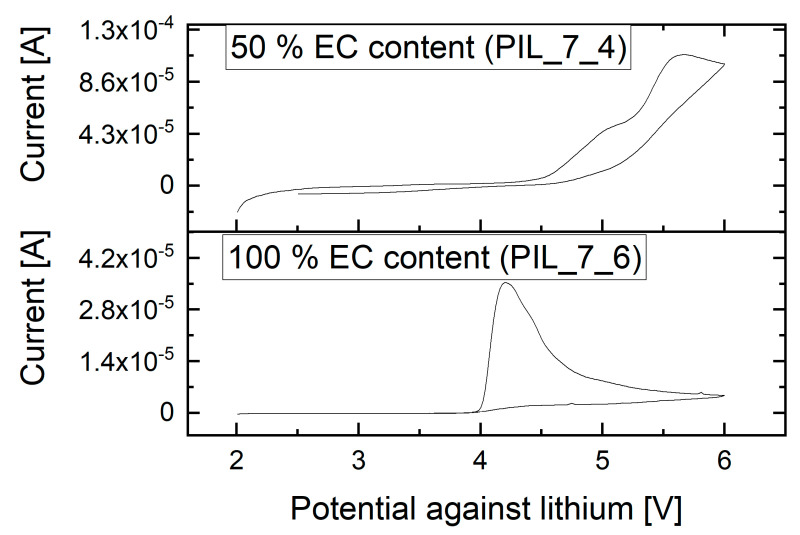
Results of the cyclic voltammetry measurements of the polymer electrolyte membranes on aluminum foil against metallic lithium at 25 °C and 1 mV·s^−1^ feed rate.

Figure 8 shows the CV measurements of the examined membranes with 50% EC and 100% EC content in the liquid electrolyte, respectively. At room temperature, the polymer electrolyte stability against lithium occurs to be sufficient for the operating range, even with NMC and lithium as electrode materials. At elevated temperatures, the electrochemical stability may decrease, resulting in side reactions. For the polymer mixture without MPPyrr-TFSI and 100% EC content, the voltage stability decreases to 4 V against lithium. The cell tests with this material are only discussed with LFP and LTO as electrode materials, since voltage range during cycling lies within the stability window.

**Figure 9:**
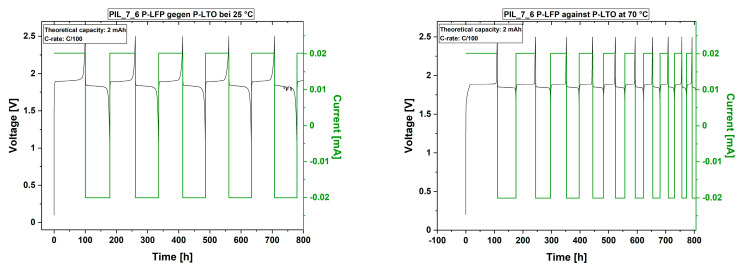
First 10 cycles of the cell test of the polymer electrolyte with 100% EC as liquid electrolyte (PIL_7_6) at 25 °C (**left**) and 70 °C (**right**) and lithium iron phosphate against lithium titanate electrodes at C/100. Images including all executed cycles are given in the Supporting material section (Figure S6).

Figure 9 shows cell tests of the PIL membrane without MPPyrr-TFSI and 100% EC as liquid electrolyte within a LFP-LTO system.

**Figure 10:**
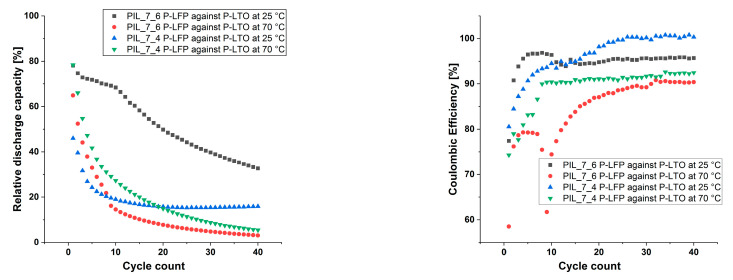
Discharge capacities relatively to the theoretical capacity of the electrode materials (**left**) and coulombic efficiencies (**right**) over the cycle count.

As the data show, the cells are stable over time. As found for the previous results, the cells continually loss capacity over the cycle count. At 70 °C the capacity loss is significantly faster as at room temperature. Further, Figure 10 shows the discharge capacities relatively to the theoretical capacity of the electrodes and the coulombic efficiencies of the cells with LFP vs. LTO in a comparative manner.

It is seen that the capacities decrease below 50% within the first 40 cycles for both membrane mixtures with varying EC content and at both studied temperatures. At 70 °C the loss in capacity proceeds faster than at 25 °C for both membrane systems. The highest capacity remains when the membrane mixture with 100% EC as liquid electrolyte was used at 25 °C. However, only the membrane with equal amounts of EC and MPPyrr-TFSI lead to a cell which built up a stable discharge capacity after several formation cycles.

Besides a stable discharge capacity, the membrane with equal amounts of MPPyrr-TFSI delivers coulombic efficiencies of 100% after several formation cycles, as Figure 10 shows. As discussed before, the two major issues remaining are the chemical stability and transference rate of lithium ions through the membrane. On one hand, a higher amount of EC seems to support the mobility of the lithium ions but reduces the chemical stability of the membrane system on the other hand. As EC is the main transport medium for the ions, a decrease of discharge capacity could be caused by decreasing amount of EC due to side reactions.

**Figure 11:**
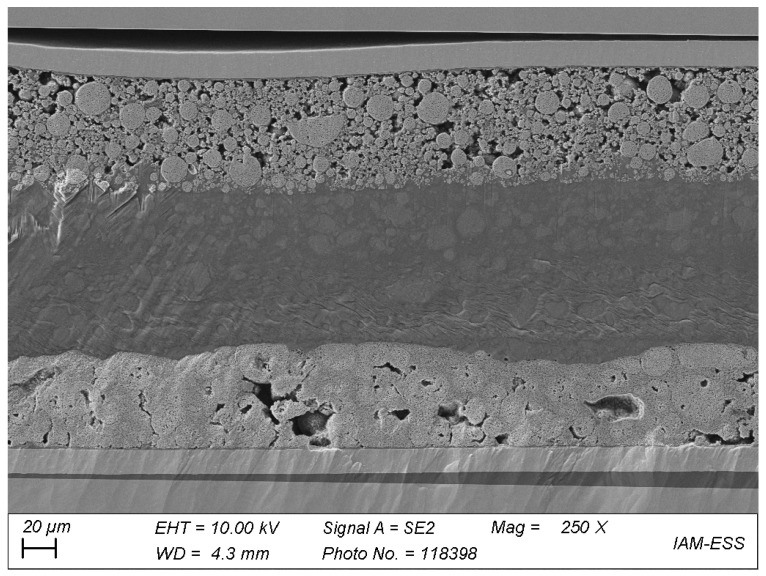
Scanning electron microscope image of the cross-section of a cell stack, post mortem after cycling at 70 °C, with PIL_7_6 coated LiFePO_4_ and Li_4_Ti_5_O_12_ electrodes; 250× magnification.

The fact that the cells did not fail in terms of a voltage breakdown, although they show strong aging effects, indicate that the mechanical aspect of the membrane is not limiting the material. This assumption was proven by scanning electron microscopy (SEM) of a post-mortem cell stack of the cell with PIL_7_6, which was cycled at 70 °C over almost 60 days. Figure 11 shows a cross-section of the stack with the LFP electrode on top of the LTO electrode.

Both electrodes were coated with the polymer and laid on top of each other before cycling in a coin cell. Within the polymer electrolyte section, no clear interface layer is seen. The two polymeric layers rather look like they have grown together. The filler particles did not sediment, which may be due to the covalent implementation within the polymeric network. A drawback, which becomes visible in the SEM analysis, is that the polymeric material is not covering the space within the active particles, especially in the LFP electrode. The monomer slurry does not funnel deeply into the pores before polymerization. Therefore, a liquid electrolyte is necessary to enable the transition of lithium ions between active material particles and the polymer electrolyte. A solvent free solution for this issue could be the preparation of special electrodes, which already feature polymer electrolyte fillers within the pores.

## 4. Conclusions

The results show that the studied polymerized ionic liquids are generally capable to build up a polymeric network that is suitable as a polymer electrolyte for lithium ion batteries. Unfortunately, the usage of a liquid electrolyte additive seems to be necessary for several reasons, e.g., the lithium ion transfer through the membrane and the wetting of active material particles. The polymers itself feature high thermal and mechanical stability and a non-crystalline appearance at room temperature. These properties make them promising candidates for safer alternatives to polyethylene separator systems and structurally superior over PEO raw materials.

As the data show, with a shift of the MPPyrr-TFSI: EC ratio towards EC, the ionic conductivity is decreasing, but the lithium ion mobility is increasing. However, the chemical stability is decreasing with increasing EC ratios. The variation of this specific liquid electrolyte composition will therefore only lead to insufficient compromises. In order to increase the performance, other electrolyte combinations and SEI forming additives will be necessary. As the data show, stable discharge capacities are possible with the PIl materials as network building matrix. The addition of additives that allow the formation of a stable SEI layer could possibly improve the electrochemical stability not just at room temperature but at elevated temperatures, as well. Strong improvements on the lithium transference through the material and in the stability of the cell chemistry are key challenges to overcome to obtain industrial relevant material properties.

## Figures and Tables

**Table 1:** wt % MPPyrr-TFSI and varying concentrations of the cross-linking agent. For this work, standard deviations are 16.7% for the determination of the ionic conductivity values. Glass transition values of complex material mixtures are only given as indications as described in the text.

PIL: P[C_2_N_A,22_]TFSI				
Liquid Electrolyte:			LiTFSI Content:	
45 wt% MPPyrr-TFSI			20 mol%	
Sample	bis-TMPTA	σ [S·cm^−1^] at 25 °C	σ [S·cm^−1^] at 60 °C	T_g_ [°C]
PIL_6_1	0.0 mol%	1.1·10^−4^	5.7·10^−4^	−52
PIL_6_5	2.5 mol%	8.7·10^−5^	6.5·10^−4^	−58
PIL_6_3	5.0 mol%	6.3·10^−5^	3.7·10^−4^	−59
PIL_6_2	10.0 mol%	6.7·10^−5^	3.8·10^−4^	−59
**PIL: P[C_8_N_A,22_]TFSI**				
**Liquid electrolyte:**			**LiTFSI content:**	
**45 wt% MPPyrr-TFSI**			**20 mol%**	
Sample	bis-TMPTA	σ [S·cm^−1^] at 25 °C	σ [S·cm^−1^] at 60 °C	T_g_ [°C]
-	0.0 mol%	-	-	-
PIL_6_7	2.5 mol%	1.3·10^−4^	7.2·10^−4^	−61
PIL_6_8	5.0 mol%	1.2·10^−4^	6.9·10^−4^	−70
PIL_6_9	10.0 mol%	9.6·10^−5^	5.4·10^−4^	−66

**Table 2:** Glass transition temperatures T_g_, ionic conductivity values σ at 25 °C and 60 °C as well as the diffusion coefficients D of ^7^Li at 25 °C in dependence of the PIL and the ratio of MPPyrr-TFSI to ethylene carbonate (EC).

Sample	PIL	Ratio MPPyrr-TFSI:EC	Amount MPPyrr- TFSI (wt %)	Amount EC (wt %)	T_g_ (°C)	σ at 25 °C (S·cm^−1^)	σ at 60 °C (S·cm^−1^)	^7^Li-D_298K_ (m^2^·s^−1^)
PIL_6_5	P[C_2_N_A,22_]TFSI	100:0	45	0	−58	8.7·10^−5^	6.5·10^−4^	2.6·10^−13^
PIL_7_1	P[C_8_N_A,22_]TFSI	100:0	45	0	−61	1.3·10^−4^	7.7·10^−4^	5.0·10^−13^
PIL_7_2	P[C_8_N_A,22_]TFSI	75:25	33.75	11.25	−57	1.3·10^−4^	6.7·10^−4^	2.3·10^−12^ 2.4·10^−11^
PIL_7_4	P[C_8_N_A,22_]TFSI	50:50	22,5	22.5	−60	1.7·10^−4^	7.9·10^−4^	2.1·10^−12^ 4.7·10^−11^
PIL_7_5	P[C_8_N_A,22_]TFSI	25:75	11.25	33.75	5 *	1.3·10^−6^	2.9·10^−5^	9.8·10^−12^ 1.8·10^−10^
PIL_7_6	P[C_8_N_A,22_]TFSI	0:100	45	0	19 *	5.8·10^−9^	7.5·10^−7^	9.9·10^−12^ 1.7·10^−10^

* Onset-temperatures of melting peaks.

## Supplementary Materials

The following are available online at https://www.mdpi.com/article/10.3390/polym13244469/s1, Figure S1: Exemplary PFG-NMR data for the PIL membranes with 45 wt% MPPyrr-TFSI and 2.5 wt% cross-linker with P[C2NA,22]TFSI (PIL_6_5) (left) and P[C8NA,22]TFSI (PIL_7_1) (right), Figure S2: Exemplary DSC-data for the PIL membranes of P[C8NA,22]TFSI with 2.5 wt% cross-linker and additional 45 wt% MPPyrr-TFSI (PIL_7_1) (left) and 45 wt% EC (PIL_7_6) (right), Figure S3: Used equivalent circuit for fitting of the EIS data, Figure S4: Exemplary EIS data and fit of a single measurement (given data in the manuscript are average results of 4 single measurements) for the PIL membranes of P[C_8_N_A,22_]TFSI with 2.5 wt% cross-linker and additional 45 wt% MPPyrr-TFSI (PIL_7_1) (left) and 45 wt% EC (PIL_7_6) (right) at 25 °C, Table S1: Overview over the compositions of the PIL membranes with 45 wt% MPPyrr-TFSI and TMPTA as cross-linkers, Figure S5: Cell test of the polymer electrolyte with MPPyrr-TFSI to EC ratio of 1:1 (PIL_7_4) at 70 °C with the electrode combinations NMC against lithium (left; C/200) and LFT against LTO (right; C/100), Figure S6: Cell test of the polymer electrolyte with 100% EC as liquid electrolyte (PIL_7_6) at 25 °C (left) and 70 °C (right) and LFP against LTO electrodes at C/100.
