# Supplementary material for: Poly(ionic liquid) Based Composite Electrolytes for Lithium Ion Batteries
Source: Polymers (Basel). 2021 Dec 20;13(24):4469. doi: 10.3390/polym13244469 (PMC8707941; doi:10.3390/polym13244469)
Supplement: Supplementary file 1 [file polymers-13-04469-s001.zip › polymers-1478450-supplementary.pdf]

polymers

MDPI

Supplementary Material

Poly(Ionic Liquid) Based Composite Electrolytes for Lithium Ion Batteries

Robert Löwe [1,2], Thomas Hanemann [1,2], Tatiana Zinkevich [1] and Andreas Hofmann [1,*]

[1] Institute for Applied Materials, Karlsruhe Institute of Technology, Hermann-von-Helmholtz-Platz 1, D-76344 Eggenstein-Leopoldshafen, Germany; robert.loewe@kit.edu (R.L.); thomas.hanemann@kit.edu (T.H.); tazink1984@gmail.com (T.Z.)

[2] Department of Microsystems Engineering, University of Freiburg, Georges-Köhler-Allee 102, D-79110 Freiburg, Germany

* Correspondence: andreas.hofmann2@kit.edu; Tel.: +49-72-160-825-920

SI-1. Additional information to the measurement methods (identical to our previous publication on polymerized ionic liquids)

"Ionic conductivity values were received from electrochemical impedance spectroscopy. For the measurements, a *Zahner Zennium* potentiostat (Zahner, Germany) and Swagelok type cells (Swagelok, Germany) located inside a *SH-261* climate chamber (Espec, USA) were applied. The spectra were recorded between 2 MHz and 10 mHz with an amplitude of 10 mV. Within the Swagelok cells, the PIL membranes were located between two non-lithium containing blocking electrodes. The membranes bulk resistance R was determined at the point where the phase difference approaches 0 . The resulting ionic conductivity values σ are calculated from the bulk resistance values R, the electrodes surface area A and the PIL membranes thickness l via the equation:

$$\sigma = \frac{1}{R} \cdot \frac{l}{A} \quad [1]$$

Both, the surface area A and the PIL membranes thickness l are determined individually for every single experiment directly after the EIS experiment was executed. Typical electrode diameters were 12.3 to 12.5 mm and measured with a sliding caliper. The thickness of the PIL membranes was quantified with a *VRZ 4* measuring sensor (Heidenhain, Germany) and was typically between 60 µm and 150 µm.

Self-diffusion coefficient measurements were performed on a Bruker NMR spectrometer, which operates at the ^{7}Li resonance frequency of 116.6 MHz. The spectrometer was equipped with a pulse-field gradient (PFG) probehead that produces linear z-gradients with strengths up to 0.3 T·cm^{-1}. Stimulated echo (STE) pulse sequence with bipolar gradients allows to avoid issues with fast T_2-relaxation in solids and the eddy currents appearing when one deals with strong gradients [2]. The diffusion parameters were optimised and reached 3 ms for the gradient duration (δ) and 100 ms for the diffusion delay (Δ). In the experiment, the signal intensity is measured as a function of the gradient strength (g) and this obeys Stejskal-Tanner equation: [3]

$$I(t) \sim I_0 \cdot exp\left(-\frac{\Delta}{T_1}\right) \cdot \exp\left(-D\gamma^2 g^2 \delta^2 \left(\Delta - \frac{\delta}{3}\right)\right)$$

Where I_0 is the initial magnetization, T_1 denotes the spin-lattice relaxation time, γ is the gyromagnetic ratio of the ^{7}Li nucleus. In order to get rid of the relaxation time dependence, all timings were kept constant through the whole pulse sequence." [4]

Citation: Löwe, R.; Hanemann, T.; Zinkevich, T.; Hofmann, A. Poly(Ionic Liquid) Based Composite Electrolytes for Lithium Ion Batteries. *Polymers* **2021**, *13*, 4469. https://doi.org/10.3390/polym13244469

Academic Editors: Meicheng Li and Rongzong Zheng

Received: 9 November 2021
Accepted: 16 December 2021
Published: 20 December 2021

Publisher's Note: MDPI stays neutral with regard to jurisdictional claims in published maps and institutional affiliations.

Copyright: © 2021 by the authors. Licensee MDPI, Basel, Switzerland. This article is an open access article distributed under the terms and conditions of the Creative Commons Attribution (CC BY) license (https://creativecommons.org/licenses/by/4.0/).

SI-2. Examination on the imperviousness of Swagelok type cells with Karl-Fischer titration

Two samples of the composite polymer electrolyte PIL_7_4, which was polymerized in bulk, were examined with Karl-Fischer titration on a Mettler Toledo *DL39* Karl Fischer titrator with *Stromboli* autosampler ofen (heated at 140 °C), which is located at a dry room (dew point: -25 °C during measurement). The material was already stored for several months over argon, but small contaminations of water are seen to be possible. The material was split into two samples of around 220 mg, respectively.

One sample was put inside a Swagelok call, that was typically used for EIS measurements and the Swagelok cell was put in a climate chamber (ambient conditions) and held at 60 °C for 5 days. The second sample was located inside an argon filled glovebox during this period of time. Afterwards, the Swagelok cell was transferred into the glovebox and both samples were sealed into the required sample containers for Karl-Fischer titration. The titration gave water contents of 179.9 ppm for the sample which was stored in the glovebox and 575.9 ppm for the sample that was located in the Swagelok cell for 5 days at 60 °C. This result supports the thesis, that water contamination can influence EIS results of samples that are built in Swagelok cells during long-term studies. In contrast to cell tests in coin cells, the Swagelok type cells are not weld shut and not specially sealed.

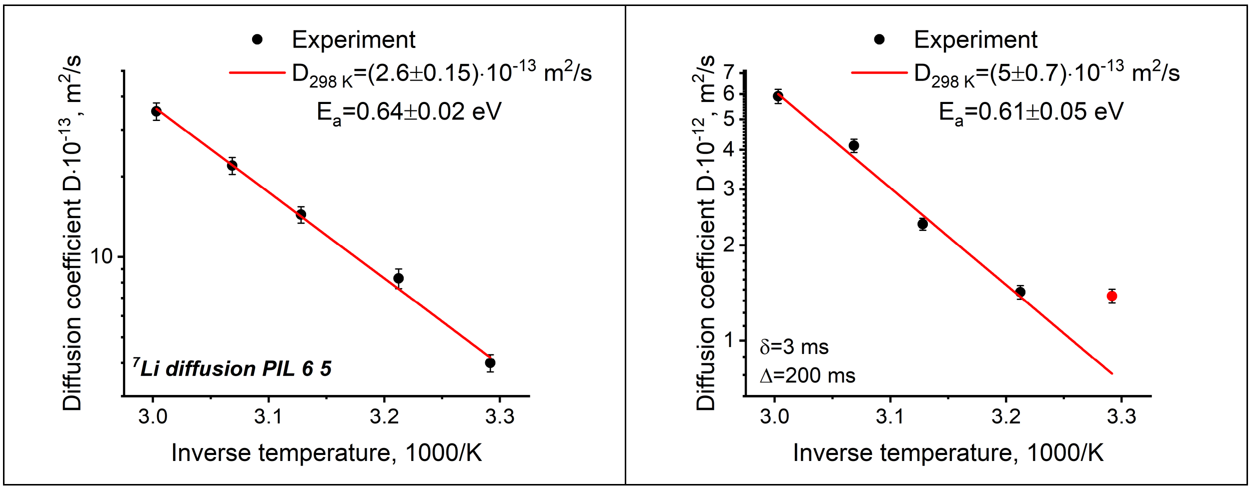

Figure S1. Exemplary PFG-NMR data for the PIL membranes with 45 wt% MPPyrr-TFSI and 2.5 wt% cross-linker with P[$C_2N_{A,22}$]TFSI (PIL_6_5) (left) and P[$C_8N_{A,22}$]TFSI (PIL_7_1) (right).

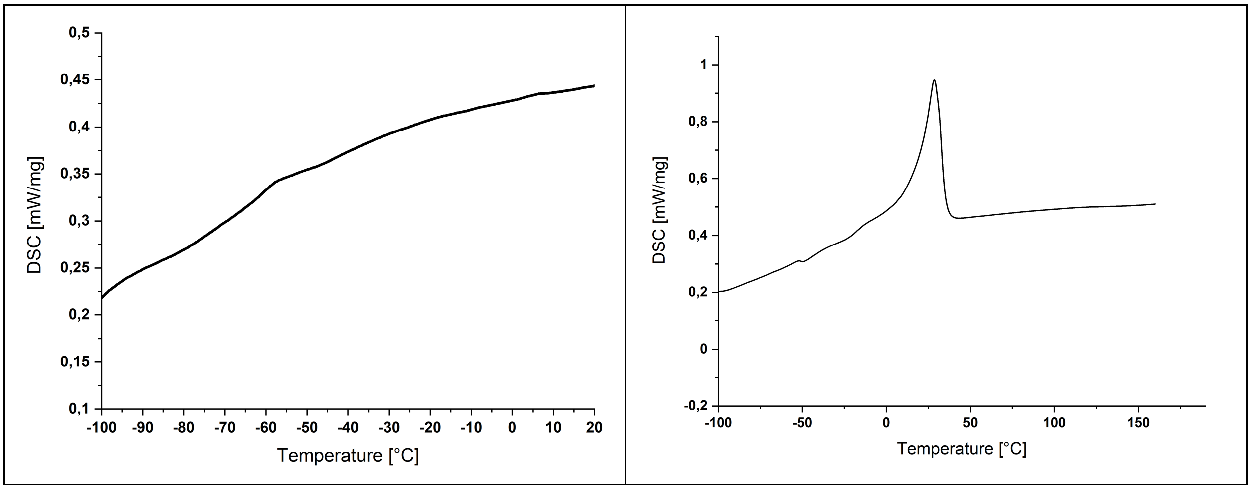

Figure S2. Exemplary DSC-data for the PIL membranes of P[$C_8N_{A,22}$]TFSI with 2.5 wt% cross-linker and additional 45 wt% MPPyrr-TFSI (PIL_7_1) (left) and 45 wt% EC (PIL_7_6) (right).

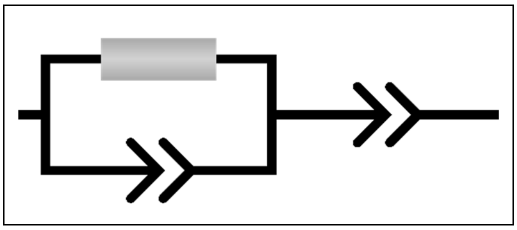

Figure S3. Used equivalent circuit for fitting of the EIS data.

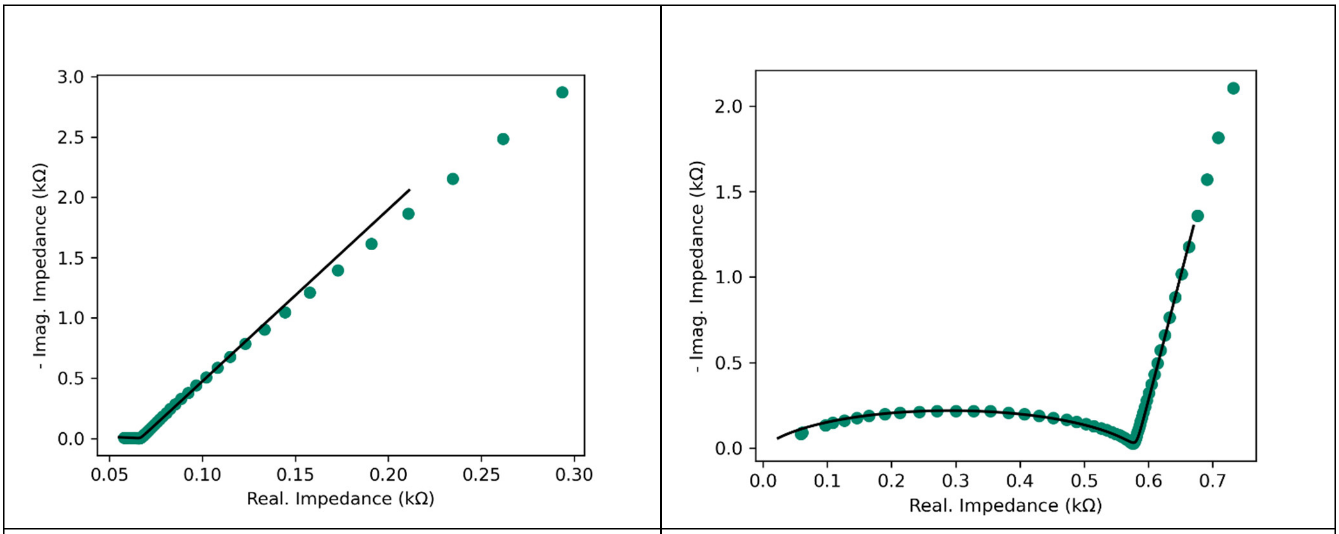

Fitting parameter:<br>Resistance 1: value: 6.68E+01 Ohm; relative error: 1.81E-01<br>CPE Q 1: value: 1.63E-07; relative error: 2.51E+01<br>CPE Alpha 1: value: 5.64E-01; relative error: 2.62E+00<br>CPE Q 2: value: 1.03E-06; relative error: 5.84E-01<br>CPE Alpha 2: value: 9.55E-01; relative error: 7.40E-02	Fitting parameter:<br>Resistance 1: value: 5.81E+02 Ohm; relative error: 7.25E-01<br>CPE Q 1: value: 5.77E-09; relative error: 1.26E+01<br>CPE Alpha 1: value: 8.23E-01; relative error: 9.42E-01<br>CPE Q 2: value: 1.62E-06; relative error: 6.07E+00<br>CPE Alpha 2: value: 9.56E-01; relative error: 9.07E-01

Figure S4. Exemplary EIS data and fit of a single measurement (given data in the manuscript are average results of 4 single measurements) for the PIL membranes of P[$C_8N_{A,22}$]TFSI with 2.5 wt% cross-linker and additional 45 wt% MPPyrr-TFSI (PIL_7_1) (left) and 45 wt% EC (PIL_7_6) (right) at 25 °C.

SI-3. Influence of membrane preparation without cross-linker

To investigate the influence of the IL MPPyrr-TFSI on the polymer layers, a composite electrolyte membrane with the following composition was prepared:

- $[C_2N_{A,22}]$TFSI to build up the polymer matrix
- Irgacure651 1 mol% with respect to $[C_2N_{A,22}]$ TFSI
- LiTFSI 20 mol% in relation to $[C_2N_{A,22}]$ TFSI
- Aerosil R7200 5 wt% in relation to the total mass of the electrolyte membrane
- MPPyrr-TFSI 45 wt% in relation to the total mass of the electrolyte membrane

After the polymerization, it was found that the electrolyte membrane is a very soft and sticky material due to the high proportion of ionic liquid. Permanent mechanical stability could not be reached. During the EIS examinations short circuits occurred regularly, especially at 60 °C, because the polymer mixture was displaced from the gap between the electrodes by the slight spring pressure inside the Swagelok cell. Therefore, the experiments had to be repeated several times until the bulk resistances could be determined. The EIS analysis of the material showed ionic conductivities of $1.1{\cdot}10^{-4}$ $S{\cdot}cm^{-1}$ at 25 °C and $5.7{\cdot}10^{-4}$ $S{\cdot}cm^{-1}$ at 60 °C. Although these values are significantly below that of the pure MPPyrr-TFSI-IL with $3.1{\cdot}10^{-3}$ $S{\cdot}cm^{-1}$ at 25 °C, it is remarkable high compared to the equivalent polymer electrolyte sample without MPPyrr-TFSI content ($5.2{\cdot}10^{-9}$ $S{\cdot}cm^{-1}$). This result confirms that the addition of non-polymerizable ionic liquids is an effective approach to increase the ionic conductivity values of the investigated ammonium PIL layer. However, due to the deficiency in mechanical stability, the use of this material combination as an electrolyte membrane in battery cells is unsuitable.

SI-4. Overview over the compositions of the PIL membranes with 45 wt% MPPyrr-TFSI and TMPTA as cross-linkers.

Table S1. Overview over the compositions of the PIL membranes with 45 wt% MPPyrr-TFSI and TMPTA as cross-linkers.

	Starting materials	$[C_2N_{A,22}]$TFSI	LiTFSI	MPPyrr-TFSI	Irgacure 651	**TMPTA**	Aerosil R7200
**PIL_6_1**	Amount per 1 mol PIL monomer [mol%]	100	20	119	1	**0**	n.a.
	Weight portion of the membrane mass [wt%]	44.5	5.3	45.0	0.2	0.0	5.0

	Starting materials	$[C_2N_{A,22}]$TFSI	LiTFSI	MPPyrr-TFSI	Irgacure 651	**di-TMPTA**	Aerosil R7200
**PIL_6_2**	Amount per 1 mol PIL monomer [mol%]	100	20	130	1	**10**	n.a.
	Weight portion of the membrane mass [wt%]	40.9	4.9	45.0	0.2	4.0	5.0

	Starting materials	$[C_2N_{A,22}]$TFSI	LiTFSI	MPPyrr-TFSI	Irgacure 651	**di-TMPTA**	Aerosil R7200
**PIL_6_3**	Amount per 1 mol PIL monomer [mol%]	100	20	124	1	**5**	n.a.
	Weight portion of the membrane mass [wt%]	42.6	5.1	45.0	0.2	2.1	5.0

**PIL_6_5**	Starting materials	$[C_2N_{A,22}]$TFSI	LiTFSI	MPPyrr-TFSI	Irgacure 651	**di-TMPTA**	Aerosil R7200

	Amount per 1 mol PIL monomer [mol%]	100	20	121	1	**2.5**	n.a.
	Weight portion of the membrane mass [wt%]	43.5	5.2	45.0	0.2	1.1	5.0

**PIL_6_9**	Starting materials	[$C_8N_{A,22}$]TFSI	LiTFSI	MPPyrr-TFSI	Irgacure 651	**di-TMPTA**	Aerosil R7200
	Amount per 1 mol PIL monomer [mol%]	100	20	148	1	**10**	n.a.
	Weight portion of the membrane mass [wt%]	42.1	4.3	45.0	0.2	3.4	5.0

**PIL_6_8**	Starting materials	[$C_8N_{A,22}$]TFSI	LiTFSI	MPPyrr-TFSI	Irgacure 651	**di-TMPTA**	Aerosil R7200
	Amount per 1 mol PIL monomer [mol%]	100	20	143	1	**5**	n.a.
	Weight portion of the membrane mass [wt%]	43.6	4.4	45.0	0.2	1.8	5.0

**PIL_6_7**	Starting materials	[$C_8N_{A,22}$]TFSI	LiTFSI	MPPyrr-TFSI	Irgacure 651	**di-TMPTA**	Aerosil R7200
	Amount per 1 mol PIL monomer [mol%]	100	20	140	1	**2.5**	n.a.
	Weight portion of the membrane mass [wt%]	44.4	4.5	45.0	0.2	0.9	5.0

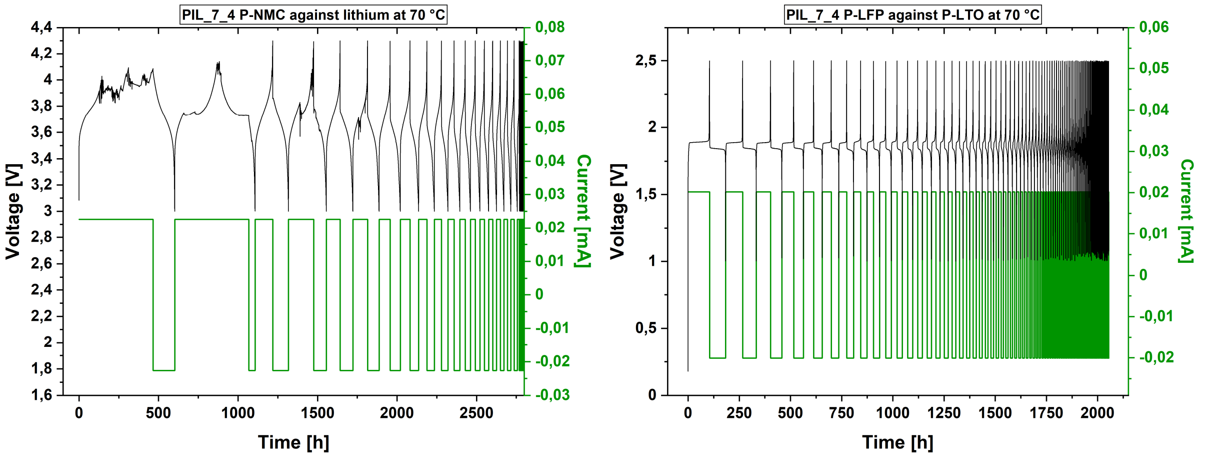

Figure S5. Cell test of the polymer electrolyte with MPPyrr-TFSI to EC ratio of 1:1 (PIL_7_4) at 70 °C with the electrode combinations *NMC against lithium* (left; C/200) and *LFT against LTO* (right; C/100).

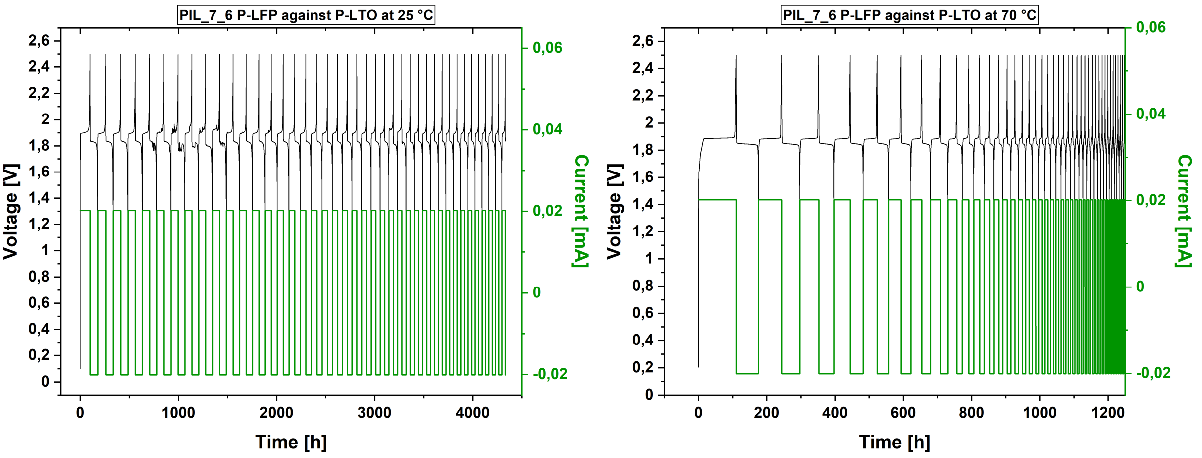

Figure S6. Cell test of the polymer electrolyte with 100% EC as liquid electrolyte (PIL_7_6) at 25 °C (left) and 70 °C (right) and LFP against LTO electrodes at C/100.

References

1. Atkins, P.W.; Paula, J. de; Bär, M. *Physikalische Chemie*, 5. Aufl.; Wiley-VCH Verl.: Weinheim, 2013, ISBN 9783527332472.
2. Tanner, J.E. Use of the Stimulated Echo in NMR Diffusion Studies. *J. Chem. Phys.* **1970**, *52*, 2523–2526, doi:10.1063/1.1673336.
3. Stejskal, E.O.; Tanner, J.E. Spin Diffusion Measurements: Spin Echoes in the Presence of a Time-Dependent Field Gradient. *J. Chem. Phys.* **1965**, *42*, 288–292, doi:10.1063/1.1695690.
4. Löwe, R.; Hanemann, T.; Zinkevich, T.; Hofmann, A. Structure–Property Relationship of Polymerized Ionic Liquids for Solid-State Electrolyte Membranes. *Polymers* **2021**, *13*, 792, doi:10.3390/polym13050792.
